# Analysis of treatment outcome variations in infantile epileptic spasms syndrome

**DOI:** 10.3389/fneur.2026.1749636

**Published:** 2026-02-17

**Authors:** Xue Gong, Jing Gan, Xiaoqian Wang, Jun Chen, Xueyi Rao, Jianjun Wang, Yajun Shen, Jia Zhang

**Affiliations:** 1 Department of Pediatrics, West China Second University Hospital, Sichuan University, Chengdu, China; 2 Key Laboratory of Birth Defects and Related Diseases of Women and Children, Sichuan University, Ministry of Education, Chengdu, China; 3 Department of Pediatrics, WCSUH-Tianfu·Sichuan Provincial Children's Hospital, Meishan, China

**Keywords:** ACTH, etiology, IESS, predictive factors, treatment response

## Abstract

**Background:**

To explore the key factors influencing outcomes in children with infantile epileptic spasms syndrome (IESS) and to elucidate their interrelationships to provide insights for optimizing clinical practice.

**Methods:**

This is a retrospective, single-center design study, included children diagnosed with IESS at West China Second Hospital of Sichuan University from April 2019 to April 2024. Descriptive analyses were performed to evaluate genetic and non-genetic etiological subgroups, categorized as structural and unknown causes. Genetic testing results were compared across groups. Pearson correlation and logistic regression analyses were employed to examine differences in treatment efficacy and identify associated risk factors.

**Results:**

In this study, 128 children diagnosed with IESS were enrolled and evenly divided into gene-positive and gene-negative groups. The gene-positive group exhibited earlier seizure onset, with a higher prevalence of hypotonia and developmental regression compared to the gene-negative group. Within the gene-negative group, children were further categorized into structural abnormality and unknown causes subgroups, among which EEG hypsarrhythmia was more frequently observed in the structural abnormality subgroup. The gene-positive group showed significantly poorer responses to ACTH, vigabatrin, and other ASMs. ACTH combined with vigabatrin therapy improved outcomes in some of the children. The non-ACTH treatment group demonstrated superior EEG improvement outcomes when compared with the ACTH-treated group (*p* = 0.028). The overall therapeutic response rate was satisfactory, with 75% in the gene-positive group and 100% in the gene-negative group. In the gene-negative group, frequent seizures and developmental regression emerged as significant risk factors for poor treatment response.

**Conclusion:**

The prognosis for IESS remains challenging, with treatment responses closely tied to etiology. Children with genetic etiologies demonstrate poorer responses to ACTH and other ASMs. However, ACTH combination with vigabatrin may improve treatment outcomes in some cases. Our findings suggest that ACTH treatment may not exert a substantial influence on long-term EEG outcomes in children with IESS.

## Introduction

Infantile epileptic spasms syndrome (IESS) is a severe, age-dependent developmental epileptic encephalopathy (DEE) characterized by epileptic spasms, developmental regression or retardation, and EEG hypsarrhythmia. The etiology of IESS is diverse; approximately 60% of cases are attributed to structural, metabolic, or infectious causes, while the remaining cases are either genetic or of unknown origin (1). The long-term consequences of IESS are profound, including intellectual disability, an increased risk of autism spectrum disorders, progression to other epilepsy syndromes, multiple refractory seizure types, and lower quality of life (1). Early diagnosis and timely interventions are critical to mitigating epileptic symptoms and preventing associated complications. However, the optimal therapeutic strategy for IESS remains unknown. Current first-line treatments for IESS typically involve steroid hormonal therapies, such as ACTH and prednisolone, or VGB. Among these, ACTH has demonstrated greater efficacy in patients without tuberous sclerosis complex (TSC) (2, 3). At our center, ACTH is the preferred initial treatment for children with IESS, with the addition of VGB considered in cases where seizure control is inadequate.

In this study, children with IESS were classified into genetic and non-genetic (i.e., structural and unknown) etiological subgroups by genetic testing, to investigate the factors contributing to differences in outcomes among children with IESS. Our aim was to elucidate the interrelationships among these factors to provide evidence-based insights for optimizing clinical practice.

## Materials and methods

### Participants

From April 2019 to April 2024, genotypic and phenotypic data were collected from children diagnosed with IESS in our pediatric neurology department, following voluntary genetic testing, which included whole-exome sequencing (WES) and copy number variation (CNV) analysis. The data encompassed demographic information (age, gender, history, family history), clinical characteristics (physical examinations, development, video electroencephalography (VEEG), brain MRI), seizure-related details (age of first seizure, frequency, and treatment history), and long-term follow-up outcomes. Nearly all patients underwent at least a basic developmental evaluation using the Griffiths Development Scales during their initial visit. Based on these assessments, developmental outcomes were categorized as: severe (DQ < 50), moderate (DQ = 50–74), or mild (DQ = 75–89). An EQ score of 90–100 was considered indicative of normal development. All patients were subsequently monitored through regular follow-up assessments to track their progress. This study was approved by the medical ethics committee of West China Second University Hospital of Sichuan University (No. 2021–138). we confirm that all methods were performed in accordance with the relevant guidelines and regulations.

**Figure 1:**
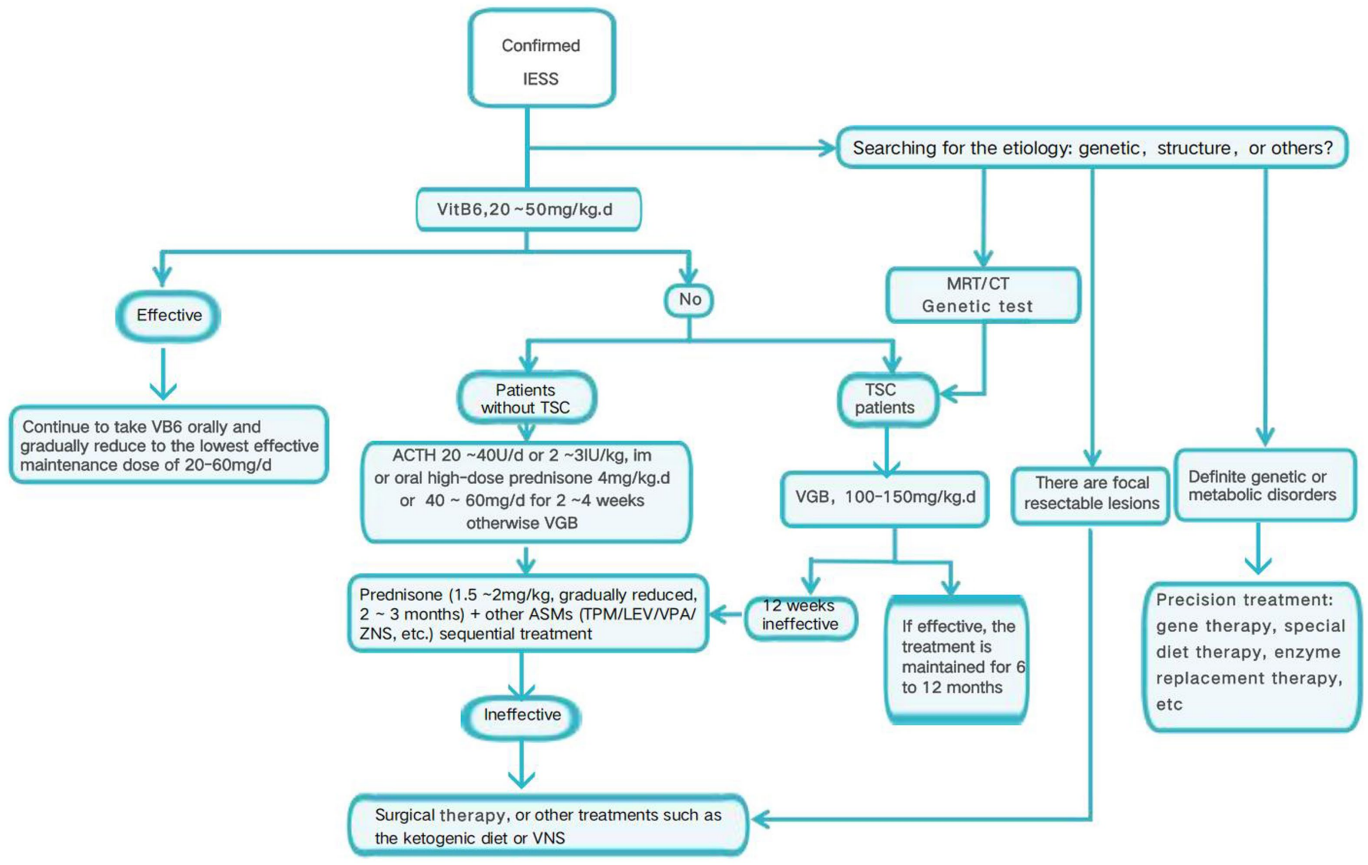
The treatment protocol for children with IESS at our center.

Patients with IESS were classified into genetic and gene-negative groups based on genetic testing results. Those with positive genetic findings consistent with the clinical phenotype were assigned to the gene-positive group, while the remaining cases were categorized as the gene-negative group. Subgroup analysis further divided the gene-negative group into structural abnormalities and unknown causes subgroups. Notably, some patients in the gene-positive group also exhibited structural abnormalities; however, due to their positive genetic test results, they remained classified within the gene-positive group. Subsequent analyses examined differences in treatment response and associated risk factors between these groups. A detailed flowchart illustrating the treatment protocol for children with IESS at our center is provided as follows (Figure 1). At our center, ACTH is the first-line treatment for children with IESS, with VGB added when seizure control remains inadequate.

## Methods

Venous blood samples from the children and their parents were collected for genetic testing including WES, CNV analysis, and multiplex ligation-dependent probe amplification (MLPA), with Sanger sequencing applied to validate potentially pathogenic single nucleotide variations (SNVs) and small fragment insertion–deletion. The pathogenicity of identified gene mutations was assessed based on clinical phenotype and American College of Medical Genetics and Genomics (ACMG) guidelines. EEG outcomes were classified as: normalized, significantly improved (> 50% reduction in discharges), improved (< 50% reduction), unchanged, or worsened. Treatment response was defined as: seizure-free (no seizures), significant effective (≥75% reduction), effective (50–75% reduction), or ineffective (< 50% reduction). Relevant data were analyzed using SPSS Version 27.0 software to investigate differences in prognosis and associated risk factors between genetic and gene-negative groups. The normality of the data sets was assessed using D ‘Agostino-Pearson and Shaphiro-Wilk normality tests. Comparisons between two groups were performed using the Mann–Whitney test (with or without Welch’s correction) or the unpaired t-test, while comparisons across multiple groups were analyzed using the Kruskal-Wallis test.

## Results

### Analysis of general information

A total of 128 children diagnosed with IESS were enrolled in this study, evenly divided into genetic (*n* = 64) and non-genetic (*n* = 64) groups. In the gene-positive group, 24 novel genotypes (37.5%) were identified. These included recurrent variants such as *STXBP1* (6 cases), copy number variations (CNVs, 2 cases), *WDR45* (2 cases), *PRRT2* (2 cases), *TSC* (2 cases), *DEPDC5* (2 cases), and single cases of *NPRL2*, *NPRL3*, *SYNGAP1*, *KCNQ2*, and *SCN1A/2A/8A*. The remaining patients harbored other rare genetic variants. Both groups had a male predominance, with 42 males (65.6%) in the gene-positive group and 38 (59.4%) in the gene-negative group. Past history, including prematurity, asphyxia, and brain injury, was significantly more frequent in the gene-positive group (16 cases, 25%) compared to the gene-negative group (7 cases, 11%, including 2 cases of HIE, *p* = 0.04). Family history, including epilepsy, febrile seizures, and mental retardation, was present in 8 cases (12.5%) in the gene-positive group and 5 cases (7.8%) in the gene-negative group, with no statistical significance (*p* = 0.4).

The age of seizure onset was statistically different between the two groups (*p* = 0.048), with a mean onset age of 6.8 months in the gene-positive group and 10.68 months in the gene-negative group, while the mean age of starting treatment was 8.2 months and 13 months, respectively. The frequency of seizures before treatment was comparable, averaging 5 per day in the gene-positive group and 6.5 per day in the gene-negative group (*p* = 0.47). Abnormal physical examinations, including head circumference size, special facial features, skin hair, muscle strength, malnutrition, and short stature, were found in 11 cases (17.2%) in the gene-positive group and 8 cases (12.5%) in the gene-negative group, without statistical significance, but hypotonia was significantly more frequent in the gene-positive group (21 cases, 32.8%) compared to the gene-negative group (3 cases, 4.7%, *p* < 0.0001). Developmental regression occurred in both groups but was significantly more pronounced in the gene-positive group (24 cases, 37.5%) compared to the gene-negative group (11 cases, 17.2%, *p* = 0.01). The general information is detailed in Table 1.

**Table 1:** General information of genetic and gene-negative groups.

Characteristics	Gene-positive group (*n* = 64)	Gene-negative group (*n* = 64)	*P* value
**Sex**			0.46
Male	42	38	
Female	22	26	
Previous history	16 (25%)	7 (11%)	0.04
Family history	8 (12.5%)	5 (7.8%)	0.4
Age of seizure	0.57y	0.89y	0.048
Seizure frequency	5 times/d	6.3 times/d	
Abnormal physical examination	11 (17.2%)	8 (12.5%)	0.47
**Muscle tone**			<0.0001
Hypotonia	21 (32.8%)	3 (4.7%)	<0.0001
Hypertonus	9 (14%)	5 (7.8%)	0.4
**Developmental retardation**			0.26
Mild	6 (9.4%)	8 (12.5%)	0.78
Moderate	14 (21.9%)	16 (25%)	0.84
Severe	36 (56.3%)	26 (40.6%)	0.21
No	7 (10.9%)	14 (21.9%)	0.4
Developmental regression	24 (37.5%)	11 (17.2%)	0.01

### Brain MRI and video electroencephalography

A total of 120 children underwent cranial MRI, revealing abnormal findings in 44 children (75.9%) in the gene-positive group and 44 children (71%) in the gene-negative group. The abnormalities observed included brain damage, neuronal migration disorders, ventricular dilatation, cerebral atrophy, and anomalies of the corpus callosum anomalies, with no statistically significant difference between the two groups (*p* = 0.68). For details, refer to the supplemental table. Among them, no obvious abnormalities were found in the ordinary head MRI of 2 cases with TSC.

All children had VEEG results obtained through regular follow-ups. Most children showed high-amplitude dysrhythmic patterns in the early stage of treatment, affecting 33 children (51.5%) in the gene-positive group and 38 children (59.4%) in the gene-negative group, with no statistically significant difference (*p* = 0.18). Additionally, pre-treatment VEEG showed severe abnormalities and frequent discharges in 48 children (75%) in the gene-positive group and 46 children (72%) in the gene-negative group, with no normal EEG patterns identified.

Follow-up VEEG assessments were performed at 3–6 months, 6–12 months, and 12–24 months after treatment initiation. At the 24-month follow-up, 8 children (19.5%) in the gene-positive group showed normalized EEG, 9 children (22%) demonstrated significant improvement, 5 children (12.2%) showed improvement, 17 children (41.5%) exhibited no improvement, and 2 children (4.9%) had worsening EEG findings, resulting in an overall improvement rate of 53.6%. In the gene-negative group, 7 children (25%) had normalized EEG, 5 children (17.8%) showed significant improvement, 4 children (14.3%) exhibited improvement, 11 children (39.3%) showed no improvement, and 1 child (3.6%) experienced exacerbation, resulting in an overall improvement rate of 57.1%. The difference in improvement rates between the two groups was not statistically significant (*p* = 0.51).

Among the gene-negative group, 21 cases with structural abnormalities were identified through cranial MRI, including neuronal migration disorders, perinatal brain injury, and post-encephalitis brain injury. No statistical differences were observed in general condition or developmental backwardness between the structural and unknown etiology groups; however, hypsarrhythmia on EEG was significantly more frequent in the structural subgroup (*p* = 0.016), as shown in Table 2.

**Table 2:** Subgroup analysis of structural lesion in gene-positive group and gene-negative group.

Gene-positive group	Structural (*n* = 30)	Non-structural (*n* = 28)	*P* value
Male/Female	23/7	15/13	0.097
Age of seizure	0.56y	0.7y	
Seizure frequency	5 times/d	5 times/d	
Dystonia	8 (26.7%)	3 (10.7%)	0.18
Developmental regression	12 (40%)	9 (32.1%)	0.59
Developmental retardation	29 (96.7%)	23 (82.1%)	0.28
Mild	2	3	0.66
Moderate	7	6	>0.99
Severe	20	14	0.29
No	1	5	0.1
EEG			
Frequent discharge	22 (73.3%)	24 (85.7%)	0.34
High amplitude EEG irregularity	19 (63.3%)	11 (39.3%)	0.11
Seizure	*n* = 27	*n* = 19	0.93
Seizure free	8	6	>0.99
Seizures reduced ≥75%	7	6	0.75
Seizures reduced ≥ 50%	5	2	0.68
Seizures reduced < 50%	7	5	>0.99

Gene-negative group	Structural (*n* = 21)	Unknown (*n* = 43)	*P* value
Male/Female	10/11	28/15	0.28
Age of seizure	0.9y	0.92y	
Seizure frequency	6.6 times/d	6.3 times/d	
Dystonia	5 (23.8%)	3 (7%)	0.1
Developmental regression	4 (19%)	8 (18.6%)	>0.99
Developmental retardation	20 (95.2%)	36 (83.7%)	0.5
Mild	4	5	0.46
Moderate	7	16	>0.99
Severe	9	15	0.59
No	1	7	0.25
EEG			
Frequent discharge	14 (66.7%)	33 (76.7%)	0.55
High amplitude EEG irregularity	17 (81%)	21 (48.8%)	0.016
Therapeutic effect	*n* = 18	*n* = 36	0.38
Seizure free	3	12	0.33
Seizures reduced ≥75%	5	12	0.76
Seizures reduced ≥ 50%	6	10	0.75
Seizures reduced < 50%	3	2	0.32

### Treatment outcome

A total of 94 children (48 in the gene-positive group and 46 in the gene-negative group) were followed for 1–5 years to assess treatment outcomes. In the gene-positive group, 15 children (31.3%) children became seizure-free, 13 (27.1%) had a ≥ 75% reduction in seizure frequency, 8 (16.7%) showed a 50–75% reduction, and 12 (25%) had <50% reduction, classified as ineffective. In the gene-negative group, 15 children (32.6%) achieved seizure-free status, 18 (39.1%) experienced a ≥ 75% reduction, 13 (28.3%) had a 50–75% reduction, and no cases were ineffective. The difference in the treatment effectiveness between the two groups was statistically significant (*p* = 0.003), driven primarily by the higher number of ineffective cases in the gene-positive group (*p* = 0.0002), while no significant differences were observed in seizure-free (*p* > 0.99), ≥75% reduction (*p* = 0.27), or 50–75% reduction groups (*p* = 0.22).

Further analysis of ACTH and vigabatrin treatment efficacy revealed a statistically significant difference in ACTH therapeutic outcomes between the two groups (*p* = 0.013), with the gene-negative group demonstrating superior responsiveness to ACTH compared to the gene-positive group. Due to the limited sample size of patients treated with vigabatrin monotherapy, no statistically significant difference was observed between the groups. However, combination therapy with ACTH and vigabatrin improved treatment efficacy in a subset of patients. Our findings indicate that the gene-negative group exhibited a higher response rate than the gene-positive group, achieving 100% efficacy with no cases of seizure exacerbation post-treatment (see Table 3 for details).

**Table 3:** The therapeutic response of ACTH and VGB in genetic and gene-negative groups.

Treatment response	Gene-positive group (*n* = 43)	Gene-negative group (*n* = 37)	*P* value
ACTH	*n* = 28	*n* = 28	0.013
Seizure free	4	7	0.5
Seizures reduced ≥75%	8	11	0.57
Seizures reduced ≥ 50%	7	10	0.56
Effective	19 (67.9%)	100%	
Seizures reduced < 50%	9 (32.1%)	0	0.002
VGB	*n* = 3	*n* = 2	
Seizure free	0	1	
Seizures reduced ≥75%	2	1	
Seizures reduced ≥ 50%	1	0	
Seizures reduced < 50%	0	0	
ACTH+VGB	*n* = 12	*n* = 7	0.66
Seizure free	4	2	>0.99
Seizures reduced ≥75%	4	3	>0.99
Seizures reduced ≥ 50%	2	2	0.6
Effective	10 (83.3%)	100%	
Seizures reduced < 50%	2 (16.7%)	0	0.5

When comparing early versus late initiation of ACTH treatment, early ACTH treatment (within one month of seizure onset) showed a higher effective rate than late treatment (after one month), but the differences were not statistically significant (*p* = 0.74, *p* = 0.2). Developmental outcomes post-treatment were also assessed, revealing an improvement in 60% of ACTH-treated children versus 70% of non-ACTH-treated children, though this difference was not statistically significant (*p* = 0.58). For details, see Table 4. Considering potential baseline differences between the two groups of infants, we further compared whether there were differences in pre-treatment EEG baseline levels between the early ACTH treatment group and the late ACTH treatment group. The results showed no statistically significant differences in frequent discharges or hypsarrhythmia on pre-treatment EEG between the two groups, with *p*-values of 0.482 and 0.82, respectively.

**Table 4:** The therapeutic response of ACTH.

Comparison items	ACTH group	Non-ACTH group	*P* value
Seizure	*n* = 67	*n* = 26	
Age of seizure	0.75y	0.74y	
Seizure frequency	6.1 times/d	4.7 times/d	
EEG			
Frequent discharge	57 (85.1%)	20 (76.9%)	0.37
High amplitude EEG irregularity	54 (80.6%)	15 (57.7%)	0.034
Therapeutic effect			
Seizure free	22	11	0.47
Seizures reduced ≥75%	24	7	0.48
Seizures reduced ≥ 50%	13	4	0.77
Effective	59 (88.1%)	22 (84.6%)	
Seizures reduced < 50%	8 (11.9%)	4 (15.4%%)	0.73
Developmental progress	40 (60%)	18 (70%)	0.58
EEG	*n* = 27	*n* = 13	0.028
Normalized	2	5	0.027
>50% reduction in discharges	6	3	>0.99
<50% reduction in discharges	2	3	0.31
Improvement	10	11	0.007
Unchanged	15	2	0.02
Worsened	2	0	>0.99

Comparison items	ACTH early treatment	ACTH late treatment	*P* value
Seizure	*n* = 43	*n* = 25	
Age of seizure	0.75y	0.75y	
Seizure frequency	6.9 times/d	4.4 times/d	
EEG			
Frequent discharge	38 (88.4%)	20 (80%)	0.48
High amplitude EEG irregularity	36 (83.7%)	18 (72%)	0.35
Therapeutic effect			
Seizure free	17	6	0.29
Seizures reduced ≥75%	16	7	0.59
Seizures reduced ≥ 50%	6	8	0.12
Effective	39 (90.7%)	21 (84%)	
Seizures reduced < 50%	4 (9.3%)	4 (16%)	0.45

Further analysis of genetic subtypes showed variable responses to treatment. Among 6 cases with *STXBP1* mutations, 2 cases became seizure-free, and 4 cases had ≥50% reduction in seizures, with treatment strategies including ACTH, VGB, topiramate, valproic acid, and ketogenic diet. Cases associated with mTOR pathway mutations (e.g., *TSC* in 2 cases, *DEPDC5* in 2 cases, *NPRL2* in 1 case, *NPRL3* in 1 case) exhibited poor outcomes, with all patients continuing to experience seizures. For *PRRT2* mutations (2 cases), oxcarbazepine achieved seizure-free status in all. *SYNGAP1* and *KCNQ2* mutations responded well to valproic acid combined with topiramate, achieving seizure-free outcomes. However, cases involving *SCN1A/2A/8A* mutations remained refractory to treatment.

We further performed a subgroup analysis on whether structural abnormalities were present in the two groups. The results showed that in the gene-positive group, children with structural abnormalities had an earlier onset of epilepsy, with an average onset age of approximately 0.56 years, and were predominantly male (about 76.7%, 23/30 cases). In contrast, children without structural abnormalities had an onset age of approximately 0.7 years, although this difference was not statistically significant. Additionally, no statistically significant differences were observed in the degree of developmental delay or treatment efficacy between children with and without structural abnormalities. In gene-negative group, the structural etiology group had an 83% effectiveness rate, with 16.7% achieving seizure-free. The unknown etiology group had a 94.4% effectiveness rate and a seizure-free rate of 33.33%. Although these differences in seizure-free rates appeared notable, the uneven sample sizes resulted in no statistically significant differences (*p* = 0.38). For details, see Table 2. Specific treatments in the gene-negative group included vitamin B6 (2 cases, both seizure-free) and levetiracetam (1 case, seizure-free). The ketogenic diet was an adjunctive therapy for 6 children in the gene-positive group, with 1 becoming seizure-free, 2 showing improvement, and 3 remaining ineffective. In the gene-negative group, 4 children received ketogenic diet treatment, with 2 becoming seizure-free, 1 showing improvement, and 1 remaining ineffective.

Developmental outcomes after treatment showed gradual improvement in 24/48 cases (50%) in the gene-positive group, with 23 cases showing no significant improvement and 1 case showing regression. In the gene-negative group, 22/38 cases (57.9%) showed gradual developmental improvement, 14 showed no insignificant improvement, and 2 cases showed regression. A further comparison of EEG outcomes between the two groups revealed statistically significant differences (*p* = 0.028), with the non-ACTH treatment group demonstrating more pronounced EEG improvement. However, there was a notable disparity in the number of cases between the two groups, see Table 4. Given the potential baseline differences between the two groups, we further compared whether there were differences in pre-treatment electroencephalogram (EEG) baseline levels between the ACTH-treated group and the non-ACTH-treated group. The results indicated no statistically significant difference in the presence of frequent discharges on pre-treatment EEG between the two groups (*p* = 0.37). However, a statistically significant difference was observed in the presence of hypsarrhythmia, with a *p*-value of 0.034.

### Treatment response correlations and logistic regression analysis

The treatment response in both genetic and non - genetic IESS groups was significantly associated with EEG outcomes, with *p* - values of 0.02 and 0.01, respectively. However, no significant correlation was found between treatment response and other factors, such as sex, age of seizure onset, abnormal physical examinations, developmental delay, frequent discharges, EEG hypsarrhythmia, cranial MRI abnormalities, seizure frequency, or the use of ACTH in the initial treatment phase.

**Figure 2:**
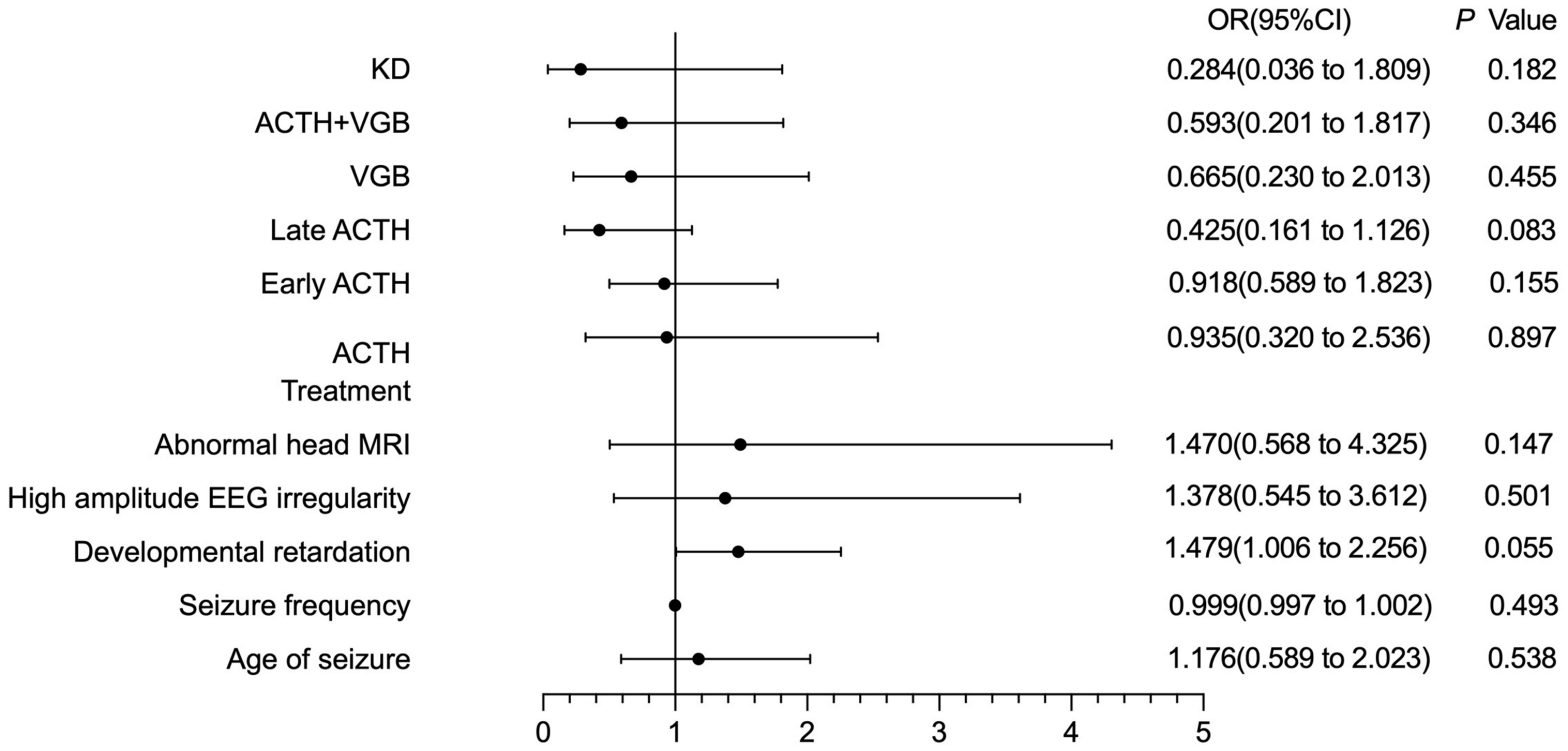
Logistic analysis of forest maps showed that prognosis in both groups was not significantly related to variables such as age of seizure onset, seizure frequency, developmental backwardness, EEG hypsarrhythmia, MRI abnormalities, or treatment factors.

Considering the differences in sample sizes across treatment outcomes, a 75% reduction in seizures was adopted as a prognostic indicator. Subsequent multifactorial logistic regression analysis revealed that, in both groups, prognosis was not significantly related to variables like age of seizure onset, seizure frequency, developmental delay, EEG hypsarrhythmia, MRI abnormalities, or treatment - related factors (including ACTH use, timing of ACTH initiation—within one month or later, VGB use, ACTH - VGB combination therapy, or ketogenic diet therapy). The overall prognostic outcomes and their correlations are illustrated in the forest plot presented in Figure 2.

## Discussion

William James West first reported his son’s disease in 1841, later termed “West’s syndrome” or “infantile spasmodic syndrome (IS),” and recently classified as “infantile epileptic spasmodic syndrome (IESS)” in the 2022 International League Against Epilepsy (ILEA) classification. IESS is a severe early-life epilepsy within the Early Epileptic Encephalopathies (EEE) group, characterized by severe, drug-resistant seizures, persistent EEG abnormalities, and developmental delays, typically presenting before 2 years of age. For patients with later onset who do not meet the diagnostic criteria for WEST syndrome, the term “epileptic spasms” is used. Recent studies have shown that the incidence of IESS is now 30.7/100,000 live births, with a male predominance (4), consistent with the 80: 48 male-to-female ratio in our study population. Generally, spasms occur between 4–9 months of age and peak around the 6th month of life, with 80–90% of cases presenting in the first year of life. However, early episodes may go unnoticed by caregivers during the initial stages of the disease. Hussain et al. reported a median treatment delay of 24.5 days among 100 IESS patients (5), whereas, in this study, the gene-positive group showed a mean onset age of 7 months with a treatment delay of 1.3 months, while the gene-negative group had a mean onset age of 10 months and a treatment delay of about 2 months.

IESS is associated with a variety of etiologies, including focal and diffuse pathologies, and the causes can be categorized as genetic, structural, infectious, metabolic, and immune abnormalities, all of which may mostly act as single causal events or in complex associations. Advances in genetic testing have revealed increasing molecular contributions to IESS. The National Infantile Spasms Association of North America found a genetic etiology in 14.4% of 161 patients with IESS, with subcategories including genetic-structural (10.0%), structural-congenital (10.8%), structural-acquired (22.4%), metabolic (4.8%), and infectious (2%) types (6), while 35% remained of unknown etiology, often associated with better outcomes (7). Similarly, in this study, the gene-negative group demonstrated superior treatment outcomes compared to the gene-positive group. In the gene-positive group, 75% achieved >50% seizure reduction, but approximately 25% showed no response. Conversely, all patients in the gene-negative group achieved >50% seizure reduction. Consider the gene-negative group, which contains children whose etiology is mostly unknown, so the prognosis is better. Because, at present, most of the IESS caused by gene variation does not have gene precision treatment, and the structural causes are also irreversible strikes, so the prognosis is naturally worse.

A recent study by Wan et al. (8) indicated that IESS patients with structural abnormalities are more likely to present with non-spasmic seizures at initial onset, and when seizures occur in early infancy, the initial response may be less favorable. In this study, a subgroup analysis was performed on whether structural abnormalities were present in the two groups of children. The results showed that in the gene-positive group, children with structural abnormalities had an earlier onset of epilepsy, at approximately 0.56 years of age, and were predominantly male (about 76.7%). In contrast, children without structural abnormalities had an onset age of approximately 0.7 years. However, no statistically significant difference in treatment outcomes was observed between the two groups of children, regardless of structural abnormalities. Within the gene-negative group, 21 cases (16.4%) had structural causes, including neuronal migration disorders, perinatal brain injury, and post-encephalitis brain injury, while 43 cases (33%) were of unknown etiology. EEG hypsarrhythmia was significantly different between these subgroups, aligning with prior single-center studies reporting EEG hypsarrhythmia in about 60% of infantile spasms (9, 10). In this study, EEG hypsarrhythmia was observed in 33 cases (51.5%) of the gene-positive group and 38 cases (59.4%) of the gene-negative group, with no statistical difference. Wirrell et al. (11) reported 133 children with IESS of unknown etiology, 85% had developmental delay. In this study, developmental delay or regression, a marker of disease progression, was reported in 89.1% of the gene-positive group and 78.1% of the gene-negative group, with 24 cases (37.5%) of developmental regression in the gene-positive group and 11 cases (17.2%) in the gene-negative group, emphasizing the influence of genetic and other etiologic factors on prognosis. Outcomes appear more favorable in gene- and structure-negative patients.

To date, more than 30 genes have been associated with IESS [1]. Boutryy-Kryza et al. (12) in a study of 73 IESS patients using array-CGH and genome sequencing, identified CNVs abnormalities in up to 15%, including *CDKL5* and *STXBP1* mutations. In this study, 24 new genotypes (37.5%) were identified in the gene-positive group, broadening the spectrum of IESS-related genes. Common variants included 6 cases of *STXBP1*, 2 cases of *CNV*, and 6 cases of *mTOR* pathway-related genes, such as 2 cases of *TSC*, 2 cases of *DEPDC5*, 1 case of *NPRL2*, and 1 case of *NPRL3*, underscoring the expanding genetic spectrum of IESS.

Recurrence of seizures is a recognized predictor of poor prognosis in IESS, often leading to intellectual disability. Early therapeutic intervention, including pyridoxine administration (150 mg), is critical to rule out pyridoxine-dependent epilepsy. At our center, almost all children in our center considered to have IESS received a high dose of vitamin B6 (20–30 mg/kg/d) as an initial treatment. While a few patients achieved seizure control with VB6 alone, most required subsequent therapies, such as ACTH. Notably, two children in our study became seizure-free on VB6 alone, though genetic testing was negative for *ALDH7A1* variants, ruling out pyridoxine dependency. Currently, steroid hormone therapy with ACTH or prednisolone, and VGB, alone or in combination, is widely accepted as first-line treatment for IESS. The combination of ACTH and VGB has shown efficacy in reducing spasms and improving EEG abnormalities. However, findings from the International Collaborative Infantile Spasms Study (ICISS) indicated that steroid hormone therapy combined with VGB did not yield superior developmental or seizure outcomes compared to steroid hormone therapy alone at 18 months. Standard first-line options of ACTH, prednisolone, or VGB remain the most commonly utilized treatments. According to existing prospective studies, ACTH is effective in 36.7–87% of cases, prednisolone in 11–70%, and VGB in 11–58% of cases (13). ACTH treatment has been associated with superior short-term responses and improved developmental outcomes, particularly in patients without a defined etiology, consistent with the findings of this study. First-line ACTH therapy has shown efficient, cost-effective, and long-term benefits in terminating IESS. Clinical trials report that 55–75% of patients treated with ACTH achieve electrical and clinical resolution within 3–14 months of follow-up, whereas fewer than half of VGB-treated patients respond.

Currently, the most widely accepted first-line treatment options for IESS include ACTH or prednisolone, and VGB, either alone or in combination. According to prospective studies, the effectiveness of ACTH ranges from 36.7 to 87%, prednisolone from 11 to 70%, and vigabatrin from 11 to 58% (1). ACTH treatment has been associated with better short-term responses and improved developmental outcomes, especially in patients without an identified etiology, which aligns with the findings of this study. ACTH is recognized as an efficient, long-term, and cost-effective approach to terminating IESS. Clinical trials have shown that 55–75% of cases treated with ACTH achieve electrical and clinical resolution at long-term follow-up (3–14 months post-treatment), while fewer than half of those treated with VGB alone show similar responses (14, 15). In this study, children with IESS were monitored regularly with EEG. Post-treatment, the EEG improvement rate was 53.6% in the gene-positive group and 57.1% in the gene-negative group, with no statistically significant difference between the two groups. These findings reinforce the effectiveness of ACTH as a primary therapeutic strategy while highlighting the need for individualized treatment plans. Further analysis of EEG outcomes between the ACTH-treated and non-ACTH-treated groups revealed a statistically significant difference (*p* = 0.028), with the non-ACTH group demonstrating more pronounced EEG improvement. However, considering the potential baseline differences between the two groups, we further compared whether there were differences in the pre-treatment electroencephalogram (EEG) baseline levels between the ACTH-treated group and the non-ACTH-treated group. The results showed no statistically significant difference in the presence of frequent discharges on the pre-treatment EEG between the two groups. However, a statistically significant difference was observed in the presence of hypsarrhythmia, with a *p*-value of 0.034. This suggests that short-term ACTH therapy may not significantly contribute to long-term EEG normalization in IESS patients. Notably, there was a considerable disparity in patient numbers between the two groups, which may be attributed to potentially lower initial seizure frequency in non-ACTH-treated patients compared to those receiving ACTH therapy.

In a study comparing steroid hormone therapy to the combination of steroid hormone therapy and VGB, the combination therapy achieved a 72% success rate in stopping spasms, compared to a 57% success rate for steroid hormone therapy alone (13). While steroid hormone combination therapy has shown promise, it carries increased risks such as VGB-associated retinal toxicity, cranial MRI changes, and possible motor deficits, necessitating further investigation before broader clinical application. A 2023 systematic review and meta-analysis, which included five randomized controlled trials (RCTs) and nine observational studies, showed that steroid hormone monotherapy significantly outperformed VGB monotherapy for new-onset IESS. Furthermore, VGB demonstrated higher efficacy in patients with TSC compared to other etiologies. However, the combination of VGB and steroid hormone therapy did not significantly improve control rates compared to steroid hormone therapy alone (16). In this study, ACTH and VGB treatment outcomes were further analyzed. Results indicated that the gene-positive group responded less favorably to ACTH compared to the gene-negative group. While the number of patients receiving VGB monotherapy was limited, the combination of ACTH with VGB increased treatment efficacy, with an efficacy rate of about 68% for ACTH monotherapy in the gene-positive group and 83.3% for the combination of ACTH and VGB.

The ketogenic diet has been proposed as an alternative treatment for IESS, showing a significant seizure reduction in about 45% of affected children (17). For patients who fail to achieve better electroclinical remission with steroid hormonal therapy and VGB, the ketogenic diet should be considered as early as possible. In this study, 10 children were added to the ketogenic diet, achieving an overall efficacy of 60%, including 3 seizure-free cases. Notably, the gene-negative group demonstrated better response rates, although the sample size was small. High doses of topiramate (10 mg /kg/d) have also shown efficacy, with 17% of patients becoming seizure-free and more than 50% experiencing seizure reduction (18). Other effective medications include valproic acid, zonisamide, and nitrazepam. For patients refractory to medication, epilepsy surgery should be considered. A 2024 systematic review and meta-analysis involving 21 studies and 531 patients showed that 69% of children became seizure-free after surgery, with a lower rate of seizure-free in genetic patients as compared to non-genetic patients (19). However, surgically curable cases of IESS in infants and children are often under-recognized, and surgical treatment in this group is often delayed or overlooked.

GulMert et al. identified etiology, age at onset, and untimely and inappropriate treatment as the most critical prognostic factors for IESS (20). The prognosis is usually worse in cases of IESS associated with severe brain malformations, infections, or genetic causes (21). Delayed treatment further exacerbates adverse outcomes.

In this study, a comparison between early ACTH treatment (initiated within 1 month of onset) and late ACTH administration revealed no statistically significant difference in treatment outcomes. Prognosis in IESS is primarily influenced by etiology rather than specific treatment protocols. Poor prognostic factors include structural etiology, preexisting developmental delays, persistent spasms, ongoing high-amplitude EEG dysrhythmias, recurrent seizures, and progression to Lennox–Gastaut syndrome. However, patients with cryptogenic IESS, representing 20–30% of patients, often have better cognitive and seizure outcomes compared to IESS as a whole (22). The study showed that 54.3% of patients with cryptogenic IESS achieve normal or near-normal developmental outcomes (23), consistent with our findings, suggesting that the gene-negative group, especially the structural-negative subgroup (i.e., the unknown etiology group) generally has better treatment and prognostic outcomes.

Multifactorial logistic analysis in this study showed no significant correlation between prognosis and factors such as age of onset, seizure frequency, developmental backwardness, high-amplitude EEG dysrhythmia, cranial MRI abnormalities, or specific treatments in either group. While etiology remains the most influential determinant of developmental outcomes in children with IESS, early treatment initiation (within 4 weeks of seizure onset) is strongly associated with better neurodevelopmental outcomes, underscoring the importance of prompt intervention to mitigate the negative effects of seizures. Consistent with prior research, structural etiology appears to be a high-risk factor for poor prognosis (22, 24, 25). In this study, the structural etiology group had an 83% treatment efficacy rate and a seizure-free rate of 16.7%, while the etiology-unknown group achieved a 94.4% efficacy rate and a 33.33% seizure-free rate. Although these differences were notable, the lack of statistical significance may be attributed to the relatively small sample size of the structural etiology group (21 cases, 16.4%) and the imbalance in case distribution between the two groups.

### Limitations

This study is limited by its retrospective, single-center design, which may introduce selection bias and limit the generalizability of the findings. Prospective multi-center studies are needed for validation. Additionally, in this study, all patients underwent whole-exome sequencing, but the testing was performed by different commercial companies, constituting a potential source of technical bias. This heterogeneity may lead to systematic under-detection or differential reporting of variants across patient subgroups, thereby introducing screening bias associated with the testing platforms. Since the study only included patients with complete genetic testing, many children with definitive acquired structural abnormalities were excluded, resulting in a relatively low proportion of structural abnormalities. Furthermore, the limited number of children treated with VGB in this study may lead to insufficient statistical power for related efficacy analyses, necessitating further validation with an expanded sample size in the future. In the long-term follow-up of this study, developmental progress was primarily assessed based on medical history and overall clinical judgment, without detailed analysis of improvements in specific skill domains. Future research may require the use of more standardized multidimensional assessment tools to obtain more refined data.

## Conclusion

The prognosis for IESS patients generally remains unfavorable. Genetic testing can yield valuable insights into the probable effectiveness of medications like ACTH in IESS. Children with genetic-cause IESS tend to respond less favorably to drugs such as ACTH than those with non-genetic IESS, but combination therapy with ACTH and VGB may enhance outcomes. ACTH therapy seems to have limited impact on long-term electroencephalographic normalization in IESS patients. Ultimately, treatment outcomes and prognosis appear to be more related to IESS etiology than to specific treatment regimens, including ACTH use, ACTH combination with VGB therapy, ketogenic diet, or other ASMs. This might be linked to the sample size of this study, and more big - data evidence is anticipated in the future.

## Data Availability

The raw data supporting the conclusions of this article will be made available by the authors, without undue reservation.
