# Correction: Analysis of treatment outcome variations in infantile epileptic spasms syndrome

**DOI:** 10.3389/fneur.2026.1857435

**Published:** 2026-05-12

**Authors:** Xue Gong, Jing Gan, Xiaoqian Wang, Jun Chen, Xueyi Rao, Jianjun Wang, Yajun Shen, Jia Zhang

**Affiliations:** 1 Department of Pediatrics, West China Second University Hospital, Sichuan University, Chengdu, China; 2 Key Laboratory of Birth Defects and Related Diseases of Women and Children, Sichuan University, Ministry of Education, Chengdu, China; 3 Department of Pediatrics, WCSUH-Tianfu·Sichuan Provincial Children's Hospital, Meishan, China

**Keywords:** ACTH, etiology, IESS, predictive factors, treatment response

In the published article, there was a mistake in Figure 1 as published. The correct Figure 1 appears below.

**Figure 1:**
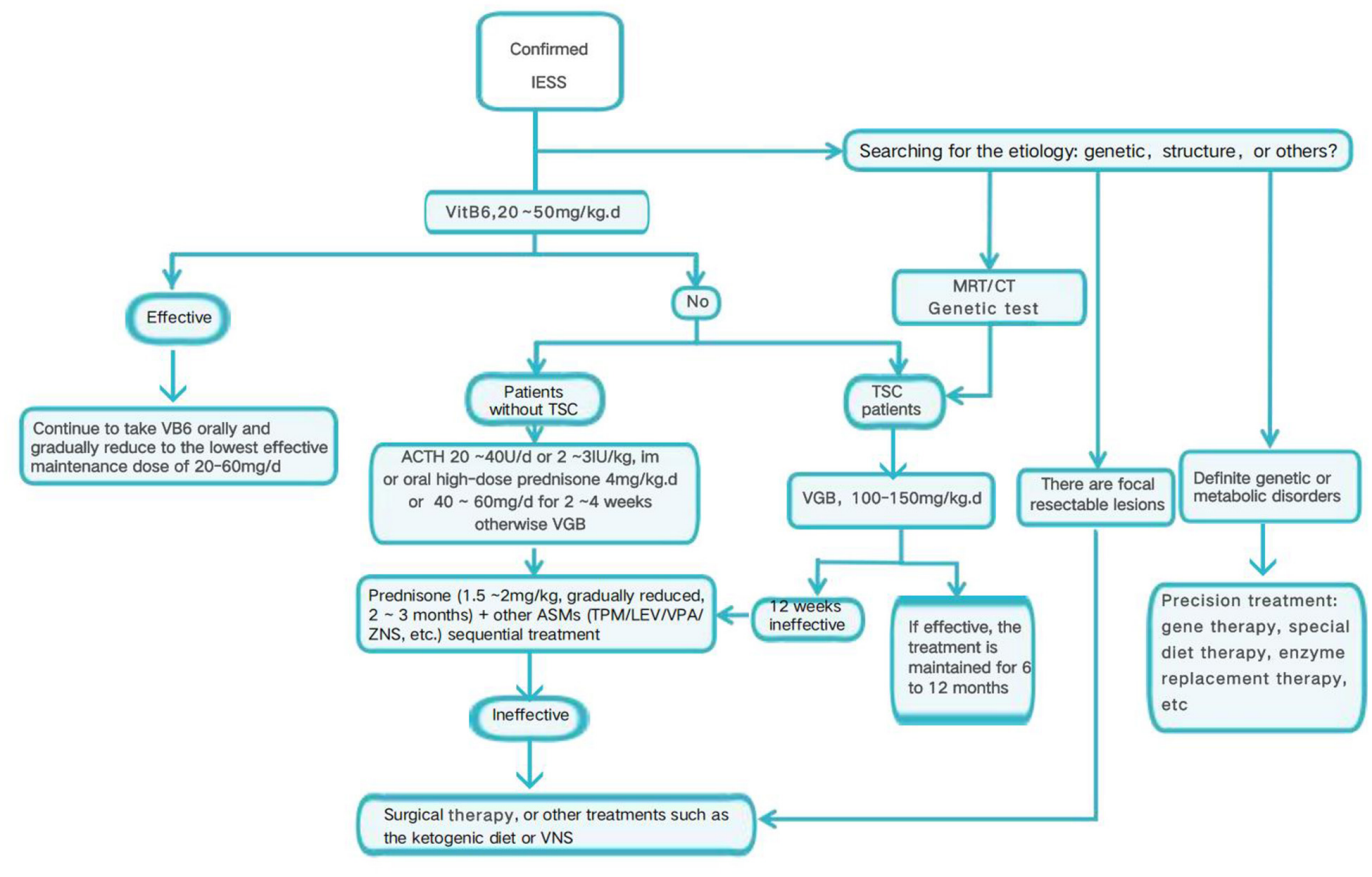
The treatment protocol for children with IESS at our center.

The text was corrected from “TSC group after 2 weeks of vigabatrin treatment ineffective” to “TSC group after 12 weeks of vigabatrin treatment ineffective”. This correction does not affect any of the study's conclusions. All statistical analyses, figures, tables, and clinical interpretations remain valid and unchanged.

The original version of this article has been updated.

